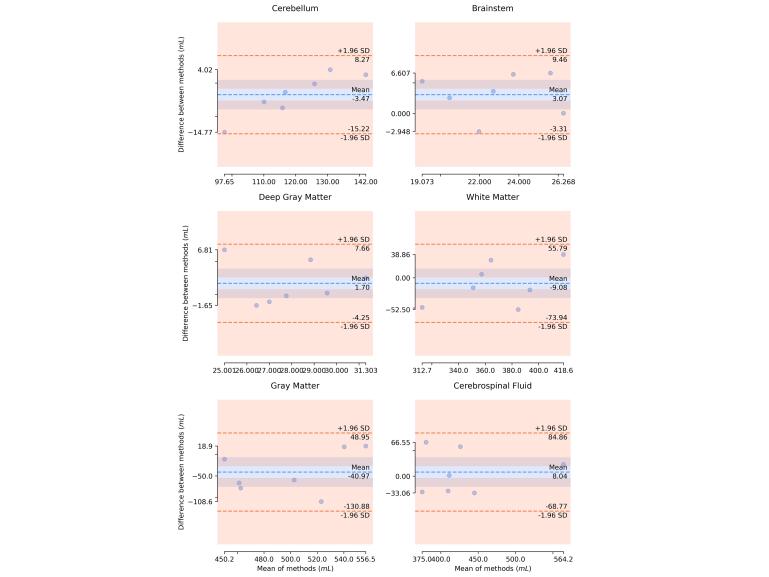# Automated multiclass tissue segmentation of clinical brain MRIs with lesions

**DOI:** 10.1016/j.nicl.2021.102769

**Published:** 2021-07-24

**Authors:** David A. Weiss, Rachit Saluja, Long Xie, James C. Gee, Leo P Sugrue, Abhijeet Pradhan, R. Nick Bryan, Andreas M. Rauschecker, Jeffrey D. Rudie

**Affiliations:** a University of Pennsylvania, United States; b University of Texas, Austin, United States; c University of California, San Francisco, United States

**Keywords:** Artificial Intelligence, Segmentation, Convolutional neural networks, Magnetic resonance images

## Abstract

Delineation and quantification of normal and abnormal brain tissues on Magnetic Resonance Images is fundamental to the diagnosis and longitudinal assessment of neurological diseases. Here we sought to develop a convolutional neural network for automated multiclass tissue segmentation of brain MRIs that was robust at typical clinical resolutions and in the presence of a variety of lesions. We trained a 3D U-Net for full brain multiclass tissue segmentation from a prior atlas-based segmentation method on an internal dataset that consisted of 558 clinical T1-weighted brain MRIs (453/52/53; training/validation/test) of patients with one of 50 different diagnostic entities (n = 362) or with a normal brain MRI (n = 196). We then used transfer learning to refine our model on an external dataset that consisted of 7 patients with hand-labeled tissue types. We evaluated the tissue-wise and intra-lesion performance with different loss functions and spatial prior information in the validation set and applied the best performing model to the internal and external test sets. The network achieved an average overall Dice score of 0.87 and volume similarity of 0.97 in the internal test set. Further, the network achieved a median intra-lesion tissue segmentation accuracy of 0.85 inside lesions within white matter and 0.61 inside lesions within gray matter. After transfer learning, the network achieved an average overall Dice score of 0.77 and volume similarity of 0.96 in the external dataset compared to human raters. The network had equivalent or better performance than the original atlas-based method on which it was trained across all metrics and produced segmentations in a hundredth of the time. We anticipate that this pipeline will be a useful tool for clinical decision support and quantitative analysis of clinical brain MRIs in the presence of lesions.

## Highlights

• A U-Net incorporating spatial prior information can successfully segment 6 brain tissue types.
• The U-Net was able to segment gray and white matter in the presence of lesions.
• The U-Net surpassed the performance of its source algorithm in an external dataset.
• Segmentations were produced in a hundredth of the time of its predecessor algorithm.

## 1 Introduction

Magnetic resonance imaging (MRI) plays a fundamental role in brain structure assessment in health and disease. Tools for automated segmentation of brain tissue volumes on MRI have been critical for research and clinical evaluation of neuropsychiatric disease (Mueller et al., 2012). However, automated segmentation of brain tissue types on clinical brain MRIs are limited due to variable acquisition parameters and the presence of lesions that distort normal anatomy and signal characteristics. By providing rapid, quantitative volumetric and morphologic information to radiologists, fully automated algorithms for brain tissue segmentation have the potential to augment the clinical workflow and improve diagnostic accuracy (Shen et al., 2017, Akkus et al., 2017). Here we developed and evaluated a rapid, automated, deep learning pipeline for segmentation of brain tissues on clinical T1-weighted MRIs in the presence of lesions from a variety of pathologies.

Semantic segmentation refers to the delineation of structures by assigning classes to every pixel or voxel in an image. For brain MRI segmentation, expectation–maximization (EM) methods (Dempster et al., 1977) have begun to replace intensity-based methods which are prone to failure due to interscan intensity inhomogeneities and distributions that do not closely match tissue intensity priors (Wells et al., 1996). However, EM techniques are stochastic, making them susceptible to local optima (Avants et al., 2011) with variable results across runs (Tustison et al., 2014). In addition such models must be fitted for each new patient and convergence can be slow (Jamshidian and Jennrich, 1997), limiting their utility in a clinical workflow where exams are read on the order of minutes to tens of minutes (McDonald et al., 2015). Deep learning offers a solution to these problems, as prediction is fast and deterministic once the model weights are learned.

Convolutional neural networks (CNNs), such as the U-Net (Ronneberger et al., 2015) and other encoder-decoder networks (Milletari et al., 2016, Badrinarayanan et al., 2017) have rapidly become state-of-the-art in biomedical image segmentation tasks. These networks consist of a downsampling arm, which reduces input images to semantic features, and an upsampling arm, which expands the features into a segmentation prediction. Recently, U-Nets have won multiple image segmentation challenges including the ischemic stroke lesions segmentation (ISLES) (Song, 2018) and multimodal brain tumor segmentation (BraTS) (Myronenko, 2018, Bakas et al., 2019) challenges. Others have developed U-Nets for tissue segmentation in nonlesional brain MRIs (Guha Roy et al., 2019, Bontempi et al., 2020, Dolz et al., 2018, Henschel et al., 2020, Zhang et al., 2021), head CT scans with and without hydrocephalus (Cai et al., 2020), and brain MRIs in the context of a variety of specific pathologies including white matter hyperintensities (Mendrik et al., 2015, Moeskops et al., 2016, Luna and Park, 2018, Chen et al., 2018, de Boer et al., 2009), multiple sclerosis (Valverde et al., 2017), and gliomas (Liu et al., 2021). However, none have been developed that are capable of segmenting brain tissue across MRIs from patients with a wide variety of underlying gray matter and white matter pathologies. If automated segmentation techniques are to be adopted into the clinical neuroradiology workflow, they will need to be able to generalize across underlying diseases and robustly recognize lesions, normal brain tissues, and the tissue type involvement of the lesion across all commonly encountered pathologies.

An important clinical distinction exists between lesions primarily involving gray matter or white matter (Rauschecker et al., 2020, Rudie et al., 2020). For example, cortical gray matter involvement is important in differentiating tumefactive demyelination from central nervous system lymphoma or glioma (Kim et al., 2009), and the location of cerebral microhemorrhages, in the basal ganglia vs. at the gray-white matter junction (Sharma et al., 2018, Blitstein and Tung, 2007), has important diagnostic implications (Martinez-Ramirez et al., 2014, Viswanathan and Chabriat, 2006). Therefore, in addition to quickly and accurately segmenting normal brain tissues on clinical MRI, we sought to develop a method that correctly segments the type of brain tissue affected by pathologic lesions [which would be complementary to other methods that segment lesions (Duong et al., 2019, Song, 2018, Myronenko, 2018, Bakas et al., 2019)].

Here we present and validate a pipeline based on a 3D U-Net for rapid segmentation of cerebrospinal fluid, cortical gray matter, white matter, deep gray matter, brainstem, and cerebellum on a large set of clinically acquired T1-weighted brain MRIs with variable acquisition parameters in patients with a wide array of brain pathologies. Because manual annotation of large datasets is prohibitively time consuming (Fischl et al., 2002) and also highly variable (Hu et al., 2020), we used a current state-of-the-art automated EM method to automatically produce segmentations for initial training. We evaluated training with different loss functions and tested whether including spatial prior information as an additional input channel improved U-Net performance. We then tested our optimal configuration on a held-out portion of our internal dataset and investigated its accuracy of segmenting the underlying tissue within lesions. Finally, we evaluated our pipeline’s performance on a smaller external dataset with whole brain manual tissue segmentations, comparing its performance to the EM method and simple registration to atlas.

## 2 Methods

### 2.1 Internal dataset

As part of an IRB approved study at our institution we retrospectively collected T1 brain MRIs from 576 patients (45 ± 14 years, 376 women) with 50 different neurologic diagnoses. T1 acquisition parameters were highly heterogeneous and included 2D-FSE and 3D-SPGR sequences. Studies were collected from 16 different scanners with scanning parameters, manufacturers and different diseases as described previously (Rauschecker et al., 2020, Rudie et al., 2020, Duong et al., 2019). A typical (mode) T1 scan in this dataset had matrix dimensions 512 × 512 × 32, 0.43 × 0.43 mm in-plane resolution, and 5 mm slice thickness. However, 58 scans had <1 × 1 mm in-plane resolution and 1 mm slice thickness and had matrix dimensions of 192 × 256 × 192. 374 of the patients had lesions characterized by abnormal hyper- or hypo-intense signal on T1 and/or T2-FLAIR weighted scans and the remaining 202 patients did not. T2-FLAIR images were also used for the purpose of manual lesion segmentation, however, the algorithm only had access to the T1 images. Pediatric patients and those with extra-axial lesions were excluded from this study.

### 2.2 External test set

We obtained an external dataset from the Grand Challenge on MR Brain Segmentation at MICCAI 2018 (MRBrainS18) [https://mrbrains18.isi.uu.nl/; (Mendrik et al., 2015). This dataset consisted of 7 patients with diabetes, Alzheimer’s disease (AD), or non-AD dementia, some of which demonstrated white matter lesions and/or chronic infarctions on their MRIs. This external dataset included manual segmentations of the following tissue types: 1) cerebrospinal fluid, 2) basal ganglia (deep gray matter), 3) white matter, 4) white matter hyperintensity, 5) (cortical) gray matter, 6) ventricles, 7) cerebellum, 8) brainstem, 9) infarctions, and 10) other. Classes 9 and 10 were excluded from our analyses because their underlying tissue type could not be clearly classified as gray or white matter.

### 2.3 Data preparation

Whole brain manual annotation of tissue types is extremely time consuming (Fischl et al., 2002). For this reason, most studies investigating 3D whole brain segmentation use automatically produced segmentations for weakly-supervised training (Guha Roy et al., 2019, Bontempi et al., 2020, Zhang et al., 2021, Fedorov et al., 2017). We similarly utilized Advanced Normalization Tools (ANTs [http://picsl.upenn.edu/software/ants; (Avants et al., 2011)), an open-source EM method, to generate labels on an internal dataset for initial training, optimization, and internal validation of our network.

For the internal dataset, we used a multimodal patch-based super-resolution technique (Manjón et al., 2010) to resample T1-weighted images to 1 mm slice thickness while leaving their in-plane resolution unchanged. Skulls were stripped and six tissue types were segmented using ANTs (Avants et al., 2011) with 0.25 (default) weighting of template-based prior probability maps. The six specific tissue types were: 1) cerebrospinal fluid (including ventricles, and subarachnoid space), 2) cortical gray matter, 3) white matter, 4) deep gray matter, 5) brainstem, and 6) cerebellum. ANTs failed in 58 (~10%) cases, of these 40 of were successfully processed after manual correction of the skull stripping step. For the other 18 no ANTs segmentation was produced, and these were excluded from the dataset. The remaining 558 subjects in the primary dataset were randomly split into training (n = 453 total; 297 with lesion[s]), validation (n = 52; 32), and test (n = 53; 33) sets. Model configuration was optimized by evaluating performance on the validation set; the top-performing model was then applied to the test set.

Input images and their targets were cropped to minimize background voxels while maintaining the entire foreground, and subsequently interpolated to 128x128x128 voxels to standardize input to the network. Input images were normalized to zero mean and unit standard deviation and transformed with random affine matrices for data augmentation before being fed into the network.

### 2.4 Automation and timing experiments

All the preprocessing steps needed to produce a U-Net segmentation from a T1-weighted image were automated with custom python programs and bash scripts. In addition to training our U-Net to segment tissues, we leveraged the same architecture to perform skull stripping to further reduce computational time. All preprocessing steps, U-Net inference, and post-process resampling to the original T1 image space were integrated to create a fully automated pipeline for brain tissue segmentation. For 25 randomly chosen cases the time taken for all preprocessing steps and U-Net segmentation was recorded and compared to the computational time required for ANTs to produce its segmentations, to assess the ability of each pipeline to produce segmentations on a clinically relevant timescale.

### 2.5 3D multiclass U-Net model

Our network was adopted from the BraTS 2017 3rd place winner (Isensee et al., 2017). We utilized the hyperparameters published alongside the architecture. The network consisted of 4 downsampling blocks followed by 4 upsampling blocks, with skip connections. Each downsampling block included a context module and each upsampling block included a localization module, as described in (Isensee et al., 2017). Kernels for all convolutional layers were 3x3x3 and both Instance Normalization and leaky ReLU activation layers followed every convolution. Outputs of the final 3 upsampling blocks were passed through 1x1x1 convolutions, resampled to match the input shape, summed, and passed through a final activation to produce C × 128 × 128 × 128 voxel segmentation volumes, where C is the number of output channels. The number of output channels and choice of activation function depended on the loss function, which we varied across experiments. When using soft Dice loss, for which sigmoid activation was used and no explicit background channel was specified (see Section 2.6), values in the prediction tensor represent the probability of a voxel belonging to a given class versus any other class, and are not normalized across channels. Voxels were assigned to the class with the highest probability, but if no label’s probability exceeded 0.5 for a given voxel, that voxel was classified as background (not brain tissue).

For training we randomly subdivided the training set into 90% training cases and 10% validation for calculating metrics after each epoch for learning rate decay and early stopping. We trained the model from randomly initialized weights using an Adam optimizer (Kingma and Adam, 2017) with an initial learning rate of 0.0005. After 10 epochs without improvement in validation loss the learning rate was decayed by a factor of 0.5. Training was stopped after 50 epochs without improvement. Training was performed using Keras 2.2.4 with Tensorflow 1.13.1 backend on an Nvidia GeForce RTX 2080Ti 11 GB GPU with a batch size of 1. The code used for model training and testing is available at https://github.com/dweiss044/multiclass_tissue_segmentation.

### 2.6 Loss function optimization

In order to determine the optimal loss function for the network we trained models using soft Dice (Milletari et al., 2016); unweighted categorical crossentropy (CCE), and median frequency weighted categorical cross entropy (WCCE) losses. For median frequency weighting experiments class weights were determined by $w_{i}=\frac{n_{b}}{n_{i}}$ where w_i_ is the weight of the i^th^ class and n_b_ and n_i_ are the median number of voxels in the background class and i^th^ class, respectively, across the entire training set. When training models with categorical crossentropy loss we utilized softmax activation at the end of the network and 7 output channels, 1 for each tissue type and 1 for background (not brain tissue), as the objective of the categorical cross entropy loss is to determine the most probable label for each voxel. When training models with soft Dice loss we utilized sigmoid activation and 6 output channels, since the objective is to maximize label-wise set similarity, which does not require an explicit background channel.

### 2.7 Spatial prior

**Fig. 1:**
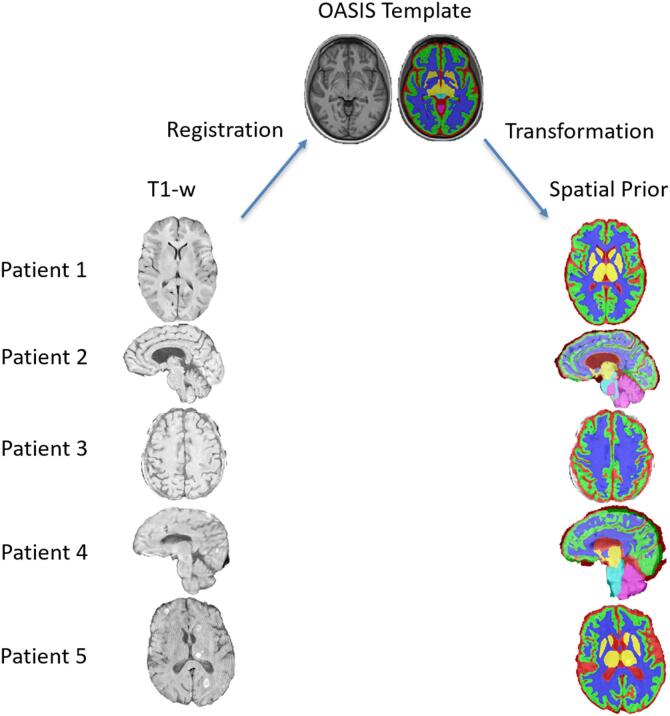
Spatial priors formed by deformable registration to template. Initial segmentations generated by transforming a template’s tissue segmentation to patient native T1 space served as patient specific spatial priors for the network. These segmentations were unable to capture the complex surfaces of gray matter and cerebrospinal fluid (see Patient 2), sometimes did not fit the cortex (see Patient 4) and resulted in overall crude segmentations.

We tested whether including spatial information from an atlas registration as a prior could improve segmentation performance in the presence of lesions and the relatively poor tissue contrast that is present on some clinical MRIs. We formed patient specific priors through a 3 step registration process of patients’ T1 image by registering the T1 image to the OASIS (Marcus et al., 2007) template beginning with a six degree of freedom rigid registration, followed by a 12 degree of freedom affine registratio, and finally a deformable registration, using ITK-SNAP’s [www.itksnap.org; (Yushkevich et al., 2006)] greedy tool. Using greedy’s label interpolation mode with σ = 0.2 vox, we applied the inverse transformations on the template’s sparse tissue segmentation to bring it into each patient’s T1 native space, creating a crude segmentation that we use as a prior (Fig. 1). The spatial prior and T1 image then formed two input channels for the U-Net.

We evaluated models trained with and without the spatial prior using the three loss functions described above on the validation set. Of the six total configurations, we chose the model that performed best on the validation set and applied it to our internal test set and the manually labeled external test set.

### 2.8 Performance metrics

We evaluated performance of the different models in this study using Dice scores, volume similarities, volume correlations, and Hausdorff Distances (95th percentile). Dice scores were given by the formula $D=\frac{2TP}{2TP+FP+FN}$, where TP, FP, and FN indicate number of true positive, false positive, and false negative voxels, respectively. Volume similarities were calculated as $\mathrm{VS}=1-\frac{{|V}_{X}-V_{Y}|}{V_{X}+V_{Y}}$, where $V_{X}$ and $V_{Y}$ represent the volumes of the true and predicted segmentations of a tissue class. Finally, Hausdorff Distances were calculated as the 95th percentile of minimum distances from all points in the predicted set to the true set of voxels for each tissue type.

### 2.9 Comparison to State-of-the-art

Our pipeline uses spatial priors to upweight spatial information for deep learning networks. We sought to validate this novel method against an nnU-Net [https://github.com/MIC-DKFZ/nnUNet; (Isensee et al., 2021), which is widely considered to be the current state-of-the-art technique in biomedical image segmentation. The nnU-Net is an automatically configuring – including preprocessing, network architecture, and post-processing – deep network for plug-and-play image segmentation. For direct comparison to our method, we trained a 3d fullres nnU-Net on our training set (453 T1 images) and tested on our internal test set (53 T1 images).

### 2.10 Intra-lesion accuracy

To assess the tissue segmentation accuracy of our method within lesions, for all validation and test set cases with lesions (n = 62), lesions were manually segmented on T2-FLAIR sequences into “gray matter lesion” and “white matter lesion” classes by JR, a neuroradiology fellow with 2 years of post-residency experience, using ITK-SNAP (Blitstein and Tung, 2007). Our aim was to assess our model’s ability to identify underlying tissue types inside of lesions, thus cortical gray matter and deep gray matter lesions were condensed into a single gray matter lesion label. Intra-lesion accuracies were calculated as the proportion of white matter lesion voxels correctly predicted as “white matter” (label 3) and the proportion of gray matter lesion voxels correctly predicted as “cortical gray matter” (label 2) or “deep gray matter” (label 4) by our network.

### 2.11 Evaluation of the external test set

We matched the manual segmentation labels of the external dataset to those of our internal dataset by condensing ventricles and cerebrospinal fluid into one class and relabeling white matter hyperintensities as white matter. The manual segmentations of the external dataset differed from those of the automated EM algorithm used to segment our internal dataset as they included more cerebrospinal fluid around the cerebellum and brainstem, and the midbrain was classified as white matter, where the EM algorithm classified the midbrain as deep gray matter (Fig. 6**B**). Therefore, we utilized transfer learning to port our model to the new dataset. To achieve this, we performed leave-one-out cross validation in the external dataset by training models initialized with the top-performing model on our internal dataset. For each fold of cross-validation, we used 5 external dataset cases for training, 1 for calculating metrics for learning rate decay and early stopping (validation), and 1 left out for testing. When performing transfer learning we utilized the same configuration as the original top performing model. Predictions for each of the 7 cases were obtained using the model for which it was left out. We compared our model’s performance to that of ANTs and segmentation by deformable registration to a standard atlas (Marcus et al., 2007), which was equivalent to using our spatial prior as segmentation.

### 2.12 Statistical testing

Two sample Z tests were used to compare different models’ Dice scores, Hausdorff distances (95th percentile), and volume similarities. Z scores were calculated as

$$Z=\frac{{\overset{-}{x}}_{1}-{\overset{-}{x}}_{2}}{\sqrt{\frac{\sigma_{1}^{2}}{n_{1}}+\frac{\sigma_{2}^{2}}{n_{2}}}}$$

where ${\overset{-}{x}}_{1}$ and ${\overset{-}{x}}_{2}$ denote the means, $\sigma_{1}$ and $\sigma_{2}$ represent the standard deviations, and $n_{1}$ and $n_{2}$ represent the sample sizes of the metric distributions of models 1 and 2, respectively. For a given metric, if Z > 2.5 then model 1 had a significantly higher average than model 2, and if Z < −2.5 then model 1 had a significantly lower average than model 2. For Dice scores and volume similarities a higher average corresponds to better performance but for Hausdorff Distances a lower average corresponds to better performance. Z scores<2.5 in magnitude were considered insignificant. Intra-lesion accuracies were compared using two-sample paired t-tests.

## 3 Results

### 3.1 Choice of loss function

**Fig. 2:**
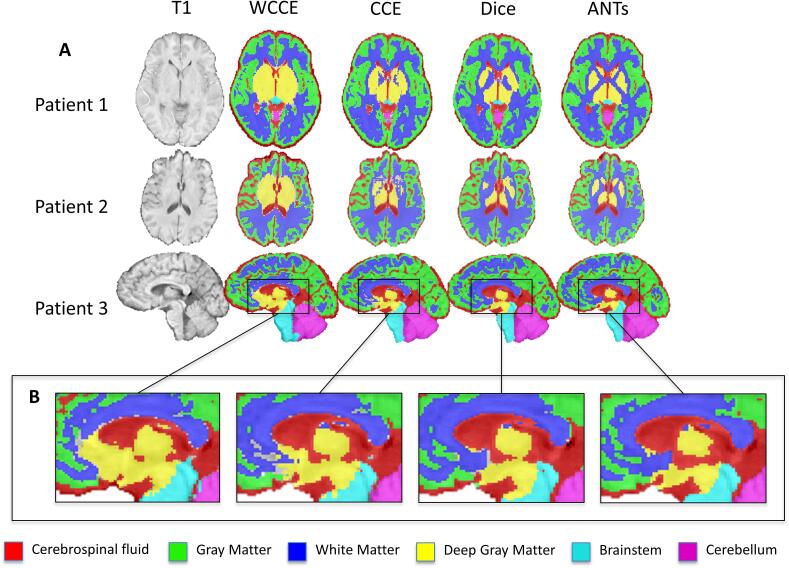
Qualitative comparisons of output segmentations for models using different loss functions and no spatial prior. (A) T1-weighted images of three patients are depicted next to multiclass segmentations resulting from WCCE, CCE, and soft Dice models with no spatial prior and the ANTs target segmentations. Low signal contrast between tissue types in patients 1 and 2 resulted in poor model performance for the deep gray matter class. (B) The WCCE model overpredicts deep gray structures, while the CCE model predicts voxels around deep gray matter structures and ventricles as background more often than the soft Dice model.

Tissue segmentations varied according to the choice of loss function. Mean Dice scores ranged between 0.65 and 0.95 for different tissue types and loss functions in the validation set (Table 1). Soft Dice and unweighted CCE losses significantly outperformed WCCE loss on overall Dice score (Z = 3.21, soft Dice; Z = 2.98, CCE) and on all tissue types except gray and white matter (Table 1). The soft Dice and CCE models both achieved an average overall Dice of 0.86 ± 0.05 in the validation set. Although their overall Dice scores were similar (Z = 0.23), the soft Dice model achieved significantly higher Dice scores in the two smallest classes, brainstem (Z = 5.89) and deep gray (Z = 2.56). The WCCE model greatly overpredicted the deep gray class, missegmenting surrounding white matter, brainstem, and cerebrospinal fluid as deep gray matter. The CCE model also tended to predict many voxels around the deep gray matter structures and ventricles as background, making the soft Dice model output appear better visually (Fig. 2**B**).

**Table 1:** Effect of loss function on network performance in the internal validation dataset. Mean ± standard deviation tissue-wise Dice scores in the validation set for models trained with soft Dice, unweighted categorical cross entropy (CCE), median frequency weighted categorical cross entropy (WCCE), with and without spatial priors, and simple registration to ATLAS are listed. *Significantly better than corresponding tissue and loss function without prior, ^x^Significantly better than WCCE, ^+^Significantly better than CCE.

		Cerebellum	Brainstem	Deep Gray	White Matter	Gray Matter	CSF	Overall
Prior	**Loss Function**							
Without	Soft Dice	0.95 ± 0.05^x^	0.90 ± 0.04^x+^	0.78 ± 0.07^x+^	0.86 ± 0.05^x^	0.81 ± 0.07	0.84 ± 0.05^x^	0.86 ± 0.05^x^
	CCE	0.94 ± 0.04^x^	0.84 ± 0.06^x^	0.74 ± 0.06^x^	0.86 ± 0.05^x^	0.81 ± 0.07	0.84 ± 0.05^x^	0.85 ± 0.05^x^
	WCCE	0.92 ± 0.04	0.81 ± 0.04	0.65 ± 0.06	0.83 ± 0.06	0.79 ± 0.07	0.80 ± 0.06	0.82 ± 0.06
With	Soft Dice	0.96 ± 0.07	0.93 ± 0.03^x^*	0.84 ± 0.09 ^x^*	0.88 ± 0.06	0.83 ± 0.07^x^	0.82 ± 0.06^x^	0.86 ± 0.06^x^
	CCE	0.96 ± 0.04^x^	0.91 ± 0.04^x^*	0.84 ± 0.08 ^x^*	0.88 ± 0.05	0.83 ± 0.06^x^	0.83 ± 0.06^x^	0.86 ± 0.05^x^
	WCCE	0.94 ± 0.04	0.84 ± 0.06*	0.77 ± 0.07*	0.86 ± 0.06	0.77 ± 0.08	0.77 ± 0.07	0.82 ± 0.06
	Registration	0.91 ± 0.08	0.84 ± 0.10	0.79 ± 0.06	0.82 ± 0.04	0.67 ± 0.06	0.58 ± 0.08	0.73 ± 0.05

### 3.2 Effect of the spatial prior on model performance

**Fig. 3:**
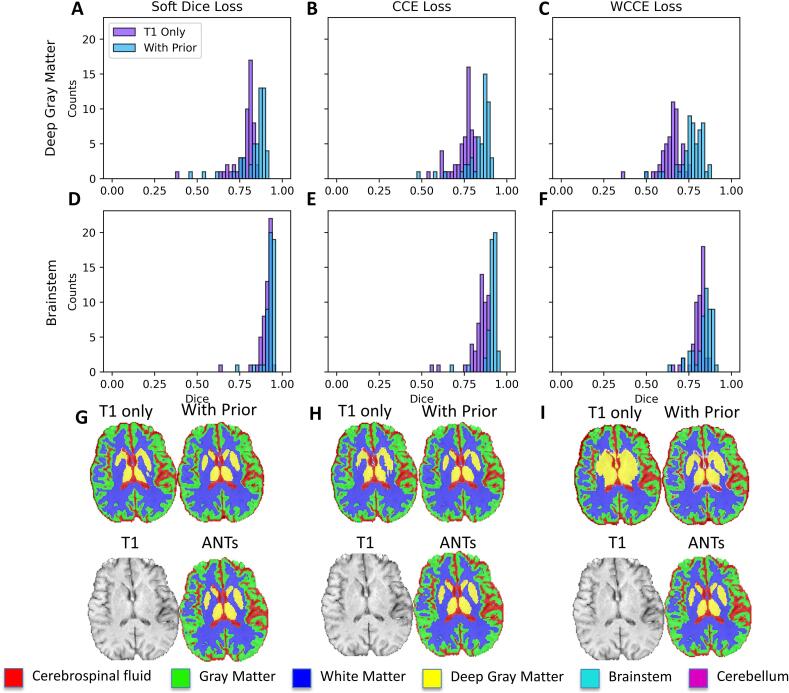
Effect of spatial prior on model performance in the validation set. Improvement in deep gray matter Dice scores as a result of adding the spatial prior can be seen for (A) soft Dice (Z = 3.48), (B) unweighted CCE (Z = 6.48), and (C) weighted CCE (Z = 9.34) models. Improvement for brainstem Dice scores as a result of adding the spatial prior were also observed for (D) soft Dice (Z = 2.98), (E) unweighted CCE (Z = 6.5) and (E) weighted CCE (Z = 3.19). The same slice of the same patient is shown for (G) soft Dice, (H) CCE, and (I) WCCE models next to ANTs targets to depict the effect of the spatial prior on deep gray segmentation. It is evident in (G), (H), and (I) that adding the spatial prior produces segmentations that more closely match ANTs in deep gray matter regions.

All models trained on only the T1 sequence had relatively low performance on the deep gray tissue class, with the soft Dice model achieving the best mean Dice of 0.78 ± 0.07. Dice scores for all other tissue classes ranged between 0.81 ± 0.07 for gray matter to 0.95 ± 0.05 for cerebellum (Table 1). The relatively worse performance for deep gray matter was largely due to low signal contrast between deep gray structures and white matter (Fig. 2**A**). After the addition of the spatial prior as an additional input channel we observed an increase in model performance in the deep gray (Z = 3.48, soft Dice; Z = 6.48, CCE; Z = 9.34, WCCE) (Fig. 3**A-C**) and the brainstem classes (Z = 2.98, soft Dice; Z = 6.5, CCE; Z = 3.19, WCCE) (Fig. 3**D-F**) for all loss functions. Differences in Dice score following the addition of the spatial prior were insignificant for all other labels across all loss functions (|Z| < 2.5).

### 3.3 Volume similarity

**Fig. 4:**
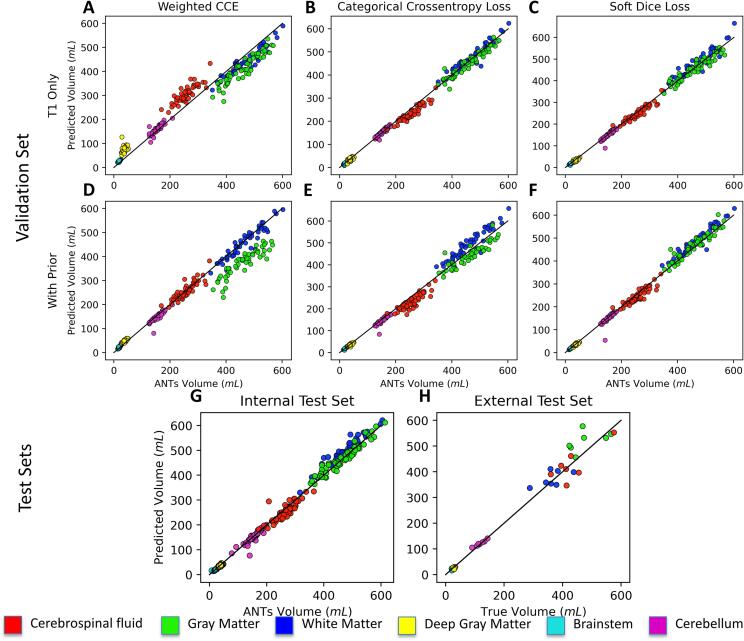
Volume similarities of tissue types in the validation and test sets. Plots of predicted to true tissue volumes are depicted for models trained with CCE, WCCE, and soft Dice losses without (A-C) and with (D-F) the spatial prior. As can be seen by relative closeness of points to the 1:1 identity line, volume similarity (VS) was greater using (F) soft Dice with spatial prior compared to (E) CCE with spatial prior for white matter (VS_Dice_ = 0.98 ± 0.01; VS_CCE_ = 0.96 ± 0.01; Z = 4.94), gray matter (VS_Dice_ = 0.98 ± 0.02; VS_CCE_ = 0.97 ± 0.02; Z = 3.45), and cerebrospinal fluid (VS_Dice_ = 0.97 ± 0.02; VS_CCE_ = 0.94 ± 0.04; Z = 4.28). High volume similarities can be seen as well in the (G) primary test set and the (H) secondary test set.

**Supplementary Fig. 1:**
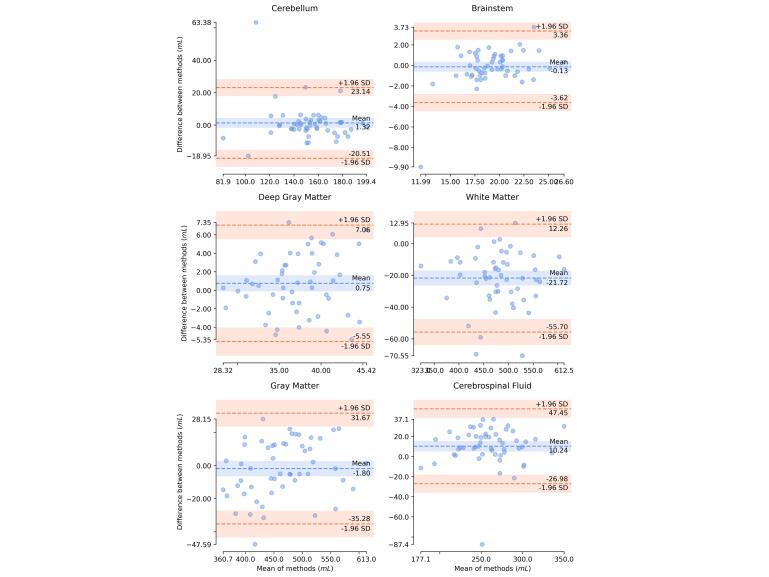


Soft Dice and CCE models with the spatial prior performed best in regards to Dice coefficients, but the CCE model tended to misclassify voxels at tissue interfaces as background, making the soft Dice model superior to the CCE model in volume similarity for white matter (Z = 4.94), gray matter (Z = 3.45), and cerebrospinal fluid (Z = 4.28) tissue types (Fig. 4**E-F,** Fig. S1-S2). Therefore, we selected the soft Dice model with spatial priors as our optimal configuration and evaluated its performance on the internal and external test sets.

### 3.4 Internal test set

**Fig. 5:**
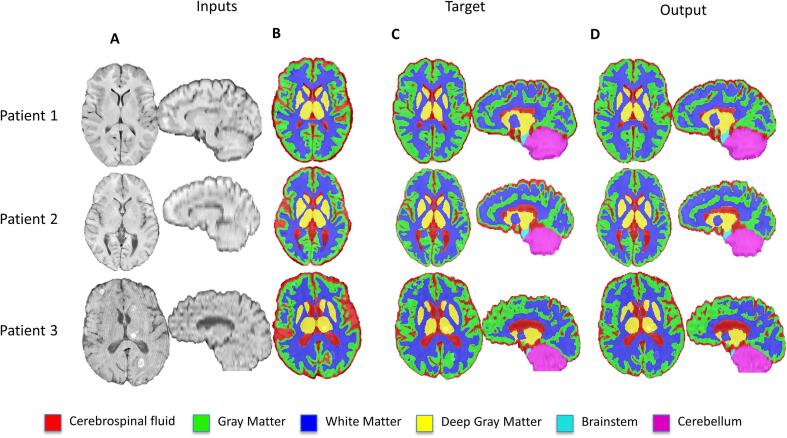
Example multiclass tissue segmentation in the internal test set. (A) T1-weighted and (B) spatial prior images are inputs to the network. (C) ANTs generated segmentations served as labels for evaluation. (D) Example segmentations by the soft Dice model with spatial prior.

The optimal model performed well at predicting tissue segmentations on the internal test set (Fig. 5). For all tissue types there was a high level of agreement between our predictions and the ANTs labels (Dice > 0.82). Mean volume similarity was above 0.96 for each tissue type and mean Hausdorff distances (95th percentile) were below 3 mm for every tissue type. Tissue-wise Dice coefficients, Hausdorff Distances (95th percentile), and volume similarities were similar to those achieved by the 3d_fullres nnU-Net. No significant differences were found between the soft Dice loss model with spatial priors and the nnU-Net (|Z| < 2.5) (Table 2).

**Table 2:** Tissue-wise performance of the soft Dice with spatial prior model in the internal test set relative to ANTs “ground truth.” Mean ± standard deviation Dice coefficients indicate high agreement with the “ground truth” across tissue types. Tissue-wise performance of the nnUNet model are reported alongside those of the soft Dice model with spatial prior. Differences between the models were not statistically significant, |Z| < 2.5 across all tissues and metrics.

		Cerebellum	Brainstem	Deep Gray	White Matter	Gray Matter	CSF	Overall
Metric	**Algorithm**							
Dice	CNN (with prior)	0.95 ± 0.06	0.91 ± 0.07	0.84 ± 0.06	0.89 ± 0.03	0.83 ± 0.04	0.83 ± 0.06	0.87 ± 0.04
	nnUNet	0.92 ± 0.09	0.90 ± 0.11	0.87 ± 0.07	0.88 ± 0.05	0.83 ± 0.08	0.79 ± 0.13	0.86 ± 0.07
Hausdorff Distance (mm)	CNN (with prior)	1.56 ± 1.51	1.41 ± 3.26	2.31 ± 4.17	0.96 ± 0.58	0.81 ± 0.42	1.16 ± 0.64	
	nnUNet	2.06 ± 4.04	1.85 ± 4.34	2.01 ± 4.37	1.05 ± 0.68	0.87 ± 0.53	1.36 ± 1.08	
Volume Similarity	CNN (with prior)	0.98 ± 0.04	0.97 ± 0.06	0.96 ± 0.02	0.98 ± 0.02	0.98 ± 0.01	0.97 ± 0.03	
	nnUNet	0.97 ± 0.06	0.96 ± 0.06	0.96 ± 0.25	0.99 ± 0.02	0.98 ± 0.02	0.97 ± 0.03	

### 3.5 External test set

**Fig. 6:**
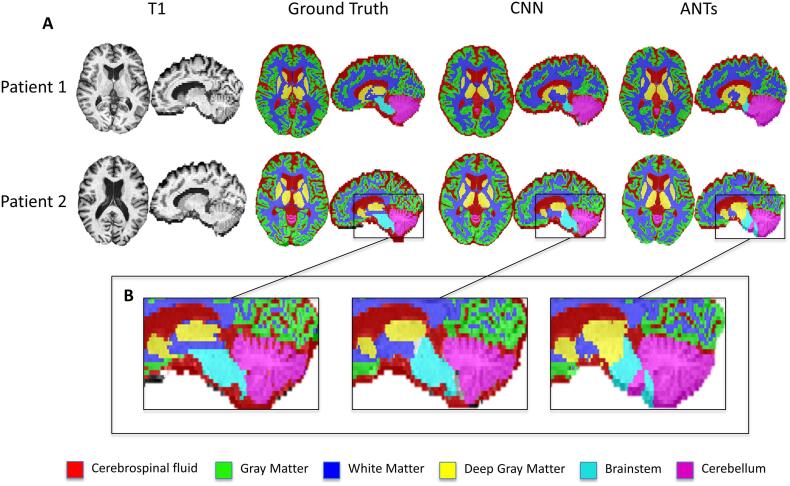
Example multiclass tissue segmentations of the external test set. (A) T1-weighted images with manual (Ground Truth) segmentations, predicted segmentations of the transfer learned U-Net soft Dice model with spatial prior, and ANTs segmentations are depicted. (B) Heuristic differences between the manual segmentations and ANTs segmentations can be seen around the midbrain-brainstem interface and the manual segmentation’s inclusion of cerebrospinal fluid around the brainstem and cerebellum.

The performance of the top-performing model and the transfer learned models (See Section 2.11) were evaluated on the external test set. When performing transfer learning initialized with the top-performing model we sought to keep the network configuration consistent. We therefore utilized a soft Dice loss function and included spatial priors as inputs to the network just as we had for the internal test set. Example segmentations of the external test set generated by transfer learned models are shown in Fig. 6. The transfer learned models achieved a mean overall Dice of 0.77 ± 0.01 and tissue-wise Dice scores ranged from 0.69 ± 0.01 for cerebrospinal fluid to 0.91 ± 0.02 for cerebellum across the 7 predictions produced by cross-validation. Label-wise performance of the original model, transfer learned models, ANTs, and registration to ATLAS are summarized in Table 3.

**Table 3:** Tissue-wise performance in the external test set. Metrics are reported for the soft Dice model before and after transfer learning to accommodate the new dataset. *Significantly better than CNN, ^x^Significantly better than ANTs, ^+^Significantly better than registration.

		Cerebellum	Brainstem	Deep Gray	White Matter	Gray Matter	CSF	Overall
Metric	**Algorithm**							
Dice	CNN	0.87 ± 0.02	0.75 ± 0.02	0.60 ± 0.03	0.81 ± 0.03	0.71 ± 0.02	0.64 ± 0.04	0.73 ± 0.01
	CNN (transfer)	0.91 ± 0.02*^x+^	0.79 ± 0.01*^x+^	0.75 ± 0.01*^x+^	0.82 ± 0.02^+^	0.77 ± 0.03*^+^	0.69 ± 0.01*^+^	0.77 ± 0.02*^+^
	ANTs	0.83 ± 0.02	0.69 ± 0.03	0.67 ± 0.03	0.83 ± 0.04^+^	0.76 ± 0.04^+^	0.66 ± 0.03^+^	0.76 ± 0.02^+^
	Registration	0.84 ± 0.04	0.71 ± 0.02	0.69 ± 0.03	0.76 ± 0.02	0.64 ± 0.03	0.57 ± 0.01	0.68 ± 0.02
Hausdorff Distance(mm)	CNN	3.58 ± 0.44	13.94 ± 3.76	6.36 ± 2.60	2.89 ± 0.10	1.68 ± 0.30	6.11 ± 0.68	
	CNN (transfer)	3.29 ± 0.64^x^	8.00 ± 1.2*^+^	4.81 ± 1.14^x^	2.77 ± 0.35	2.04 ± 0.31	2.90 ± 0.48*^x+^	
	ANTs	6.71 ± 0.92	12.37 ± 6.36	9.54 ± 1.82	2.46 ± 0.46^+^	1.91 ± 0.34	10.5 ± 1.76	
	Registration	4.01 ± 0.74^x^	13.46 ± 3.06	4.82 ± 0.61^x^	2.94 ± 0.07	1.95 ± 0.09	7.90 ± 0.61^x^	
Volume Similarity	CNN	0.93 ± 0.03	0.89 ± 0.06	0.96 ± 0.04	0.94 ± 0.04	0.93 ± 0.04	0.94 ± 0.05	
	CNN (transfer)	0.98 ± 0.02*^x+^	0.91 ± 0.05	0.95 ± 0.05^x+^	0.96 ± 0.02^+^	0.95 ± 0.04	0.96 ± 0.03^x+^	
	ANTs	0.85 ± 0.03	0.94 ± 0.04	0.83 ± 0.05	0.92 ± 0.03	0.96 ± 0.04	0.86 ± 0.05	
	Registration	0.86 ± 0.05	0.90 ± 0.05	0.82 ± 0.05	0.88 ± 0.04	0.94 ± 0.04	0.86 ± 0.06	

Our model achieved higher Dice than ANTs in cerebellum (Z = 6.9), brainstem (Z = 9.2), and deep gray matter (Z = 6.1) classes and higher Dice than registration to ATLAS in all tissue types. Hausdorff Distances (95th percentile) of the model were lower than those of ANTs for cerebellum (Z = −8.0), deep gray (Z = −5.8), and cerebrospinal fluid (Z = −11.0), and lower than those of registration to atlas for brainstem (Z = −4.4) and cerebrospinal fluid (Z = −17.0). Volume similarities for the model were higher than both ANTs and registration to atlas in cerebellum (Z = 5.1; Z = 7.4), deep gray (Z = 4.7; Z = 3.87), white matter (Z = 3.7; Z = 2.2), and cerebrospinal fluid (Z = 3.8; Z = 4.1).

### 3.6 Intra-lesion accuracy

**Fig. 7:**
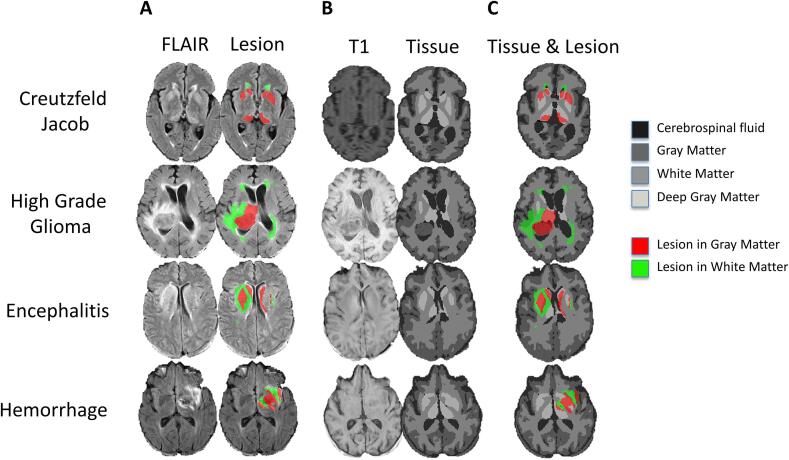
Tissue segmentation in the presence of lesions in the primary test set. (A) Original FLAIR image (first column) and manual segmentation of lesions in gray matter (red) and in white matter (green) (second column). (B) Original T1 image (first column) and tissue segmentation using the soft Dice model with spatial prior (second column). (C) Lesions manually segmented by underlying tissue type are depicted overlaid on multiclass tissue segmentations. Lesions in white matter were well classified as white matter by the U-Net (median accuracy = 0.85) and lesions in gray matter were often classified as gray matter or deep gray matter (median accuracy = 0.61). (For interpretation of the references to colour in this figure legend, the reader is referred to the web version of this article.)

The soft Dice model was the best performing model overall. This model functioned well even in the presence of large lesions that altered the signal and spatial configuration of tissue types (Fig. 7). Using the soft Dice model with spatial prior, median intra-lesion accuracy, μ_1/2_, across the internal validation and test sets was 0.61 (IQR = 0.25) for gray matter lesions and 0.85 (IQR = 0.17) for white matter lesions. The model achieved higher intra-lesion accuracy with the spatial prior than without for white matter lesions (μ_1/2_ = 0.83, IQR = 0.25; t = 4.1, p = 0.0001, paired *t*-test), but differences were insignificant for gray matter lesions (μ_1/2_ = 0.57, IQR = 0.24; t = 1.48, p = 0.14, paired *t*-test) (Table 4).

**Table 4:** Accuracy of gray matter and white matter segmentations within lesions. Average intra-lesion accuracies (μ) are reported ± standard deviations and median intra-lesion accuracies (μ_1/2_) are reported with interquartile ranges (Q1-Q3) for the U-Net. With the spatial prior the model does significantly better for white matter lesions (p = 0.0001; paired *t*-test) but has no significant difference for gray matter lesions (p = 0.14; paired *t*-test).

		Intra-lesion Gray Matter	Intra-lesion White Matter
Metric	**Model**		
μ	With Prior	0.60 ± 0.21	0.83 ± 0.14
	Without Prior	0.57 ± 0.22	0.77 ± 0.17
μ_1/2_	With Prior	0.61 (0.45–0.71)	0.85 (0.77–0.94)
	Without Prior	0.57 (0.44–0.68)	0.83 (0.66–0.90)

### 3.7 Computational time

Steps to produce segmentations using our pipeline were timed for a random subset of 25 cases. Preprocessing, including registration to template, transforming the spatial prior to patient T1 space, cropping background, resampling volumes to 128x128x128 voxels, z-score standardizing, and loading Keras models was completed in 78.03 ± 16.88 sec (μ ± σ). Skullstripping was completed in 4.12 ± 0.08 sec (μ ± σ) and U-Net tissue segmentation inference took 1.65 ± 0.09 sec (μ ± σ). Combining preprocessing and inference steps, our fully automated method produced tissue segmentations in 83.80 ± 16.86 sec (μ ± σ) for these 25 cases. In comparison, ANTs produced tissue segmentations in 4698.14 ± 1443.79 sec (μ ± σ) for the same 25 cases. This corresponds to a reduction in computational time by our pipeline of two orders of magnitude.

## 4 Discussion

Traditional brain tissue segmentation methods are computationally intensive, slow, and prone to inaccuracy when applied to brain MRIs with lesions (Battaglini et al., 2012, Valverde et al., 2015, Dadar and Collins, 2021). While recent use of deep neural networks has significantly improved speed, no existing networks work with heterogeneous clinical brain MRIs with the variety of pathologies encountered in clinical neuroradiology practice. Fast, automated full-brain tissue segmentation capable of performing the presence of diverse brain pathology could provide diagnostically relevant quantitative information about tissue involvement and volumes of brain tissue that could be integrated into clinical workflow (Rauschecker et al., 2020, Rudie et al., 2020). Here, we developed a fully automated pipeline that utilized spatial prior information and a 3D U-Net for simultaneous segmentation of 6 tissue types on T1-weighted brain MRIs of patients with a wide array of brain pathologies. This pipeline achieved equivalent performance to the nnU-Net, which is widely accepted as state-of-the-art in biomedical image segmentation, across all tissue-wise performance metrics. Further, our method produced segmentations in a hundredth of the time of its source algorithm and surpassed the algorithm with which it was trained in segmentation performance when compared to manual segmentations in an external dataset.

Because of the variability of tissue contrast, resolution, and presentation of lesions in clinical brain MRIs, the inclusion of a spatial prior to our model facilitated learning of neuroanatomical structures and stabilized network outputs. Our internal dataset consisted of studies collected from 16 different scanner types with variable acquisition parameters at typical clinical resolutions. Many of the T1 images in this dataset had low resolution and poor signal contrast between tissue types, especially between deep gray matter structures and white matter. CNNs learn spatial features around units’ receptive field sizes (Liu et al., 2020), with deeper layers learning larger, more complex – global – features as their receptive fields grow relative to the size of the original image with each downsampling operation. In contrast, early layers learn local features based on local contrast variation. As such, CNNs are highly signal dependent. We hypothesize that the addition of a spatial prior through a deformable atlas registration greatly improved the network’s performance in small and low signal contrast classes and resulted in qualitatively and quantitatively improved segmentations, as it allowed the network to learn global spatial information more easily.

The spatial prior also allowed for more accurate tissue segmentation in the presence of lesions. CNNs use a mixture of spatial and intensity features to achieve accurate segmentation (Moeskops et al., 2016), so the inclusion of additional spatial information helped the network in regions of signal abnormalities. Lesions in our primary dataset were variable in size, shape, intensity, location, and were indicative of vastly different underlying pathologies. Inclusion of the spatial prior significantly increased white matter intra-lesion accuracy (p = 0.0001) but had insignificant effect on the gray matter intra-lesion accuracy (p = 0.14) of the model. This difference is likely due to lesions presenting as hypointensities on T1, making white matter lesions appear more like gray matter based on intensity alone, meaning that the network was better able to localize the lesions when equipped with the prior spatial information. It should be noted, though, that not all lesions can be clearly delineated as white matter or gray matter, as some lesions consist of entirely different tissue types, such as hemorrhages, abscesses, and tumors, which were represented in our dataset. Our current model is ill-equipped to deal with exogenous tissue types; however, with the addition of a new “exogenous tissue” class and appropriate training segmentations a U-Net could be trained to recognize lesions that are neither gray matter, white matter, or CSF.

Before transfer learning to the out-of-sample MRBrainS18 external dataset, our pipeline achieved higher Dice scores than StarNEt and FreeSurfer (Sendra-Balcells et al., 2020). After transfer learning to the external test set with manual segmentations the U-Net significantly outperformed both ANTs and registration to template in tissue-wise Dice coefficients, Hausdorff distances (95th percentile), and volume similarities (See Table 4). Target segmentations of the external test set were heuristically different from those of the internal test set in that they included cerebrospinal fluid surrounding the brainstem and cerebellum, as well as between the sulci of the cerebellum, while the ANTs labels of our internal dataset did not. Further, the deep gray matter in our internal dataset labels included the midbrain, while the manual segmentations of the external dataset did not. These differences in segmentation targets resulted in relatively low performance in early iterations of the network. Only once we applied transfer learning with a subset of the secondary dataset did the U-Net surpass ANTs, which highlights that deep networks are constrained by the domain of their training data. The training data of our primary dataset is diverse in scanner manufacturer, field strength, and scanning parameters, but the model can be further generalized by adding multisite data, which will be requisite for integration into clinical workflows across institutions.

While other deep learning methods require less preprocessing (Bontempi et al., 2020, Dolz et al., 2018), or produce tissue segmentations in less time than ours (Guha Roy et al., 2019, Bontempi et al., 2020), these methods (Henschel et al., 2020, Zhang et al., 2021, Sendra-Balcells et al., 2020, Tushar et al., 2019) were developed for and tested in healthy subjects or patients with diseases that do not present with focal brain lesions that alter signal and distort anatomy. More recently methods have been developed and tested in patients with a single pathology (Mendrik et al., 2015, Moeskops et al., 2016, Luna and Park, 2018, Chen et al., 2018, de Boer et al., 2009, Cai et al., 2020, Valverde et al., 2017, Liu et al., 2021). In contrast, our method was developed using a wide variety of gray and white matter pathologies. We demonstrated improved segmentation performance and vastly reduced compute times compared to the widely used ANTs expectation–maximization segmentation method which was used to train our model within an external test set. Further, expectation–maximization techniques are prone to failure, as demonstrated by the ~ 10% error rate of ANTs in our internal dataset, while the U-Net did not fail in any cases. Because of this reliability, speed of inference, and overall segmentation performance, the multiclass brain tissue segmentation pipeline described in this study has the potential to be integrated into a clinical workflow and provide quantitative information about normal and abnormal brain tissues.

## 5 Conclusions

A fully automated pipeline based on a U-Net trained on labels generated from a widely used open-source algorithm was able to rapidly and accurately segment cerebrospinal fluid, cortical gray matter, white matter, deep gray matter, brainstem, and cerebellum in normal and abnormal T1 brain MRI in a hundredth of the time taken by the source algorithm. The U-Net was positively influenced by enforcing a spatial prior, included as an additional input channel to the network for training and inference. Tissue segmentation, including all preprocessing steps, was completed on average in ~ 2 min by the pipeline, representing a two orders of magnitude reduction in compute time compared to its predecessor, an expectation–maximization technique. The quality and speed of this deep learning segmentation pipeline should allow for its integration into clinical workflows.

## CRediT authorship contribution statement

**David A. Weiss:** Conceptualization, Methodology, Investigation, Software, Writing - original draft. **Rachit Saluja:** Software. **Long Xie:** Software, Writing - review & editing. **James C. Gee:** Resources. **Leo P Sugrue:** Writing - review & editing. **Abhijeet Pradhan:** Resources. **R. Nick Bryan:** Resources. **Andreas M. Rauschecker:** Conceptualization, Writing - review & editing. **Jeffrey D. Rudie:** Conceptualization, Supervision, Resources, Writing - review & editing.

## Figures and Tables

**Supplementary Fig. 2:**